# Gene flow at major transitional areas in sea bass (*Dicentrarchus labrax*) and the possible emergence of a hybrid swarm

**DOI:** 10.1002/ece3.406

**Published:** 2012-11-08

**Authors:** Nolwenn Quéré, Erick Desmarais, Costas S Tsigenopoulos, Khalid Belkhir, François Bonhomme, Bruno Guinand

**Affiliations:** 1 Institut des Sciences de l'Évolution de Montpellier, CNRS-UMR 5554, Université Montpellier 2cc63, 34095, Montpellier Cedex 5, France; 2 Station Méditerranéenne de l'Environnement Littoral2 Avenue des chantiers, 34200, Sète, France; 3 LabEx CeMEB, Université Montpellier IIplace E. Bataillon, cc63, 34095, Montpellier Cedex 5, France; 4 Hellenic Center for Marine Research, Institute of Marine Biology and GeneticsPO Box 2214, Gournes Pediados, 71500, Heraklion, Crete, Greece

**Keywords:** Hybrid swarm, introgression, microsatellite, minisatellite, sea bass, tension zones

## Abstract

The population genetic structure of sea bass (*Dicentrarchus labrax*) along a transect from the Atlantic Ocean (AO) to the Eastern Mediterranean (EM) Sea differs from that of most other marine taxa in this area. Three populations (AO, Western Mediterranean [WM], EM) are recognized today, which were originally two allopatric populations. How two ancestral genetic units have evolved into three distinct units has not been addressed yet. Therefore, to investigate mechanisms that lead to the emergence of the central WM population, its current status, and its connectivity with the two parental populations, we applied 20 nuclear loci that were either gene associated or gene independent. Results confirmed the existence of three distinct gene pools, with higher differentiation at two transitional areas, the Almeria-Oran Front (AOF) and of the Siculo-Tunisian Strait (STS), than within any population. Significant linkage disequilibrium and heterozygote excess indicated that the STS is probably another tension zone, as already described for the AOF. Neutrality tests fail to reveal marker loci that could be driven by selection within or among metapopulations, except for locus *DLA0068*. Collectively, results support that the central WM population arose by trapping two tensions zones at distinct geographic locations of limited connectivity. Population assignment further revealed that WM individuals were more introgressed than individuals from the other two metapopulations. This suggests that this population might result from hybrid swarming, and was or is still seeded by genes received through the filter of each tension zone.

## Introduction

It is well established that, despite the apparent connectivity of marine habitats and the high dispersal capabilities of marine organisms, populations may nevertheless be organized into well-defined genetic units (Hellberg et al. 2002; Hauser and Carvalho 2008; Hellberg 2009; Sanford and Kelly 2011). If present-day processes can contribute to patterns of differentiation (Galindo et al. 2006; Gerlach et al. 2007; Selkoe et al. 2008, 2010), recognized phylogeographic barriers shared by a wide range of species represent the main barriers to gene flow in the marine environment (e.g.*,* Benzie 1999; Barber et al. 2000; Patarnello et al. 2007; Galarza et al. 2009; Kelly and Palumbi 2010).

Nevertheless, nearly all marine organisms have sporadic opportunities to cross almost all physical barriers and to exchange genes between phylogeographically differentiated populations. Opportunities for any neutral or unconditionally favorable mutation to invade most of – or the whole – the species range frequently occur after secondary contacts, so gene pools should be homogenized on either side because minimal gene migration easily overcomes mutation and drift over generations. Genetic differentiation persisting across phylogeographic barriers probably indicates nonneutral limitations to gene flow, as exemplified by numerous marine hybrid zones worldwide (e.g., Gardner 1997; Strelkov et al. 2007; Hobbs et al. 2009; Gagnaire et al. 2011). The classical “porous” (or “semipermeable”) genome model states that differentiation would be wiped out from most genomic regions, except around loci conferring fitness advantage to one of the populations or, on the contrary, loci acquired during isolation and involved in endogenous incompatibilities of the Dobzhansky–Muller type (e.g., Barton and Hewitt 1985; Mallet 2005; Payseur 2010; Bierne et al. 2011). Such postzygotic barriers contribute to natural selection against hybrid F_1_, F_2_, and backcrosses (Nosil et al. 2005; Burton et al. 2006; Marshall et al. 2010), and may reveal costs of adaptation for immigrants (Hereford 2009). They, hence, contribute to the establishment of so-called tension zones reflecting the conflicting effects of dispersal of parental forms and selection against hybrids (e.g., Barton and Hewitt 1985). As the porous genome model implies wide heterogeneity in the distribution of differentiation along chromosomes, estimation of population divergence will depend on the loci investigated, their position relative to sites under selective pressure, and recombination rate (see, e.g., Bierne 2010; Payseur 2010). This leads to different introgression patterns among (classes of loci) across tension zones (e.g., Barton and Hewitt 1985). Conversely – but considerably less studied – new fit hybrid genotypes may arise from successfully recombinant individuals (Harrison 1993; Edmands et al. 2005; Hwang et al. 2011; Willett 2012). A new population (or taxon), designed as a hybrid swarm, can then emerge and successfully occupy a portion of the available habitat (Moore 1977; Arnold 1993; Jiggins and Mallet 2000).

The European sea bass *Dicentrarchus labrax* L. is a model for evolutionary studies among marine fishes, in both wild and cultured populations (Volckaert et al. 2008; Chatain and Chavanne 2009). Over its distribution area, the species is structured into three main metapopulations (Atlantic, Western, and Eastern Mediterranean [EM]), separated by well-defined barriers to gene flow (Benharrat et al. 1983; Naciri et al. 1999; Bahri-Sfar et al. 2000; Lemaire et al. 2005). Genetic homogeneity predominates in Atlantic and Western Mediterranean (WA) basins (e.g., Garcia de León et al. 1997; Naciri et al. 1999; Fritsch et al. 2007; Coscia and Mariani 2011), while the less well-studied EM basin is more structured (Bahri-Sfar et al. 2000; Castilho and Ciftci 2005). The genetic transitions over the range are each coincident with an oceanic front (the Almeria-Oran front [AOF] and the Siculo-Tunisian Strait [STS]). This may reflect tension zones that have been trapped there, with low dispersal rates and somewhat reduced population densities (Barton's trapping hypothesis; discussed in Bierne et al. 2011). Evidence that these transitional areas are tension zones in sea bass has however only been documented for the AOF by Lemaire et al. (2005). Authors demonstrated differential introgression at mtDNA and nuclear markers, but also among anonymous nuclear DNA markers (microsatellites) at the AOF. Lemaire et al. (2005) also reported significant linkage disequilibrium (LD) at population samples located close to the AOF, which are classical features of tension zones (Barton and Hewitt 1985).

All studies on sea bass have been based on a limited number of anonymous microsatellites of unknown genomic location. Therefore, the objectives of this study were to use distinct classes of nuclear markers that were either associated or independent of genes, in order to: (1) provide better estimates on the levels of differentiation at major transitional areas; (2) establish connectivity and introgression patterns for each group of markers or patterns shared by markers irrespective the group they belong to; and (3) improve knowledge on the origin of the central WM population of sea bass, in particular whether it is a hybrid swarm.

## Materials and Methods

### Samples and molecular analysis

Sixteen natural populations (*N* = 474) were sampled among the three phylogeographic units of *D. labrax* distribution area: Atlantic Ocean (AO), WM, and EM basins (Table 1). Most samples have been already analyzed in previous studies (Table 1). Nineteen microsatellite and one minisatellite markers were used, classified into two classes of markers: gene-associated loci (hereafter GAL) and gene-independent loci (GIL). A marker was classified as a GAL when it was situated either inside the span of an annotated gene or at <2 kilobases (kb) from one extremity of a coding sequence (CDS). The limit of 2 kb was retained because it generally encompasses the proximal promoter of most genes in vertebrates. Proximal promoters are known to be under functional constraints and, together with their closeness from CDS, mini- and microsatellite loci they contain may have greater probability to be influenced by selection (e.g., Li et al. 2004; Gemayel et al. 2010). A symmetrical size limit of 2 kb was also retained after the 3′-end of the CDS while – as far as we know – documented functional constraints are less reported on this side of genes. Those limits are, however, operating limits to classify GAL and GIL; they do not mean that loci located farther from CDS cannot be impacted by selection (and vice versa). The GAL comprises one mini- and three microsatellite markers that have been specifically designed for their association with growth hormone (GH: *μGH* and *mGH*), somatolactin (SL: *μSL*), and insulin-like growth factor (IGF-1: *μIGF-1*; Quéré et al. 2010; description and conditions herein) and seven microsatellite loci from two simple sequence repeat (SSR)-enriched genomic libraries (Tsigenopoulos et al. 2003; Chistiakov et al. 2004) that appeared to be close to CDS afterward. The GIL class contains nine loci originating from the same SSR-enriched genomic libraries than GAL (Table 2).

**Table 1:** Samples used in this study

Basin	Abbreviation	Population	Country	Number of individuals	Geographic location		Origin of sample
AO (31)	BB	Bay of Biscay	France	31	45°44′19″N	1°25′11″O	Fritsch et al. (2007)
WM (365)	GOU	La Goulette	Tunisia	30	36°50′06″N	10°19′59″E	Guinand et al. (2008)
	ICH	Ichkeul	Tunisia	49	37°10′21″N	9°39′55″E	Bahri-Sfar et al. (2000)
	MAR	Marsala	Italia	24	37°48′09″N	12°25′11″E	Naciri et al. (1999)
	ANN	Annaba	Algeria	24	36°54′47″N	7°46′36″E	Naciri et al. (1999)
	SBD	Sabaudia	Italia	16	41°19′36″N	13°59′30″E	Lemaire et al. (2000)
	FIUM	Fiumicino	Italia	22	41°51′36″N	12°70′02″E	Lemaire et al. (2000)
	OR	Etang de l'Or	France	54	43°34′35″N	4°01′40″E	This study
	PER	Pérols	France	30	43°33′50″N	3°58′10″E	This study
	LUN	Lunel	France	30	43°36′05″N	4°05′12″E	This study
	SET	Sète	France	38	43°23′06″N	3°42′19″E	Guinand et al. (2008)
	MUR	Murcia	Spain	48	37°51′50″N	0°43′31″O	Lemaire et al. (2005)
EM (98)	SYR	Syria	Syria	32	35°34′50″N	35°31′36″E	This study
	CYP	Cyprus	Cyprus	15	34°33′03″N	32°58′36″E	Boutet et al. (2008)
	SYRA	Syracuse	Italia	26	37°47′07″N	18°48′27″E	This study
	SEL	Selinunte	Italia	25	36°50′53″N	13°34′07″E	This study

AO, WM, and EM indicate the basin from where samples originate (AO: Atlantic Ocean; WM: Western Mediterranean; EM: Eastern Mediterranean). Numbers of individuals considered in each basin are given in brackets. References of published work that already used individual samples are reported.

**Table 2:** Microsatellite loci used in this study, including size ranges, GenBank accession numbers, fluorochrome labeling, and PCR primers

Locus	Category	Annotation	Motif	GenBank Accession	Size range (bp)^1^	Fluorochrome		Primers (5′⇒3′)
*DLA0041*	GIL	–	(TC)_8_(AC)_21_AA(AC)_3_AA(AC)_3_	DQ363864	167–201	FAM	F	AAAAGGAACAGCCCTCCAC
							R	AGCATTGTTCTTCTGAGTGACC
*DLA0044*	GAL	Unknown	(TC)_18_TTTT(TC)_6_CTCC	DQ363867	105–129	HEX	F	TCCGCTCCGCACCGAGTGAC
							R	ACCGCCCAAGGGTTGGACTG
*DLA0051*	GAL	MAPK3^a^	(GT)_16_	DQ363874	149–181	ROX	F	AGGTTCTTGGCCTGGGAATC
							R	AGTGACAGCAGCCTCCAGAG
*DLA0060*	GAL	Bestrophin 3^b^	(CA)_12_(TA)_3_AA(CA)_2_	DQ363883	119–131	FAM	F	GAGAGTTCATCCTGTTCGCTC
							R	TGTAGTAATAATGCGCTCTGCAA
*DLA0061*	GIL	–	(TG)_14_	DQ363884	153–167	FAM	F	AAAGGCCAGTGAAACTCATGT
							R	CTCCCTGTCCATCTGTCCTC
*DLA0066*	GIL*	–	(AG)_22_	DQ363887	135–161	PET	F	GTTGACCGGAGTCCTAGC
							R	GGCCATATGTGTCTTGCTT
*DLA0068*	GIL	–	(CA)_7_CGCACG(CA)_3_	DQ363889	247–266	NED	F	CAACACCTGTTCCTCTGAACC
							R	GCATTAGCATTGATTGTCCTG
*DLA0070*	GAL	Unknown	(AC)_30_	DQ363891	126–155	VIC	F	TCTGCTTGCATCTGTGGAAT
							R	GCCATCTGGCTAGCTTCACT
*DLA0073*	GIL*	–	(CT)_36_	DQ363894	157–181	NED	F	CATGACTTCATGTGCTAATGTCC
							R	AGTTCAGAGCGGCAACTGT
*DLA0075*	GIL	–	(CA)_15_	DQ363896	180–186	PET	F	CACATACACAAGCTTAACCC
							R	GGCAGAGATGGGAAATAGACA
*DLA0078*	GAL	MAN2A1^c^	(AG)_29_	DQ363899	223–243	VIC	F	AAGACTGGACCTCTGGAGACC
							R	CACAAGGAACCGAGACAAGA
*DLA0081*	GAL*	PPP2R5A^d^	(CA)_16_	DQ363902	202–222	PET	F	GACGAAGACTTCAGACGAGCTAT
							R	ATACCGAGCGACCATGTTG
*DLA0086*	GIL	–	(AC)_26_	DQ363907	191–205	FAM	F	GCTAGAGGATTCATGTCGCTT
							R	ACCTGGTGATTGGCAATTCT
*DLA0089*	GAL	LLGL1^e^	(GT)_15_	DQ363910	127–135	NED	F	ACGAGTAATGAGGACCCA
							R	GTCAAAACAGCCCACCTA
*DLA0096*	GIL*	–	(TG)_16_	EF471091	256–270	ROX	F	AACTTAGTGAAGTAACTTGTGGCAA
							R	TCGATGCATCTAGGACAGGA
*DLA0097*	GIL	–	(GT)_4_GC(GT)_2_GC(GT)_13_	EF471092	216–240	HEX	F	GCTGCAGGAGTGTGAGAGG
							R	GCGAGAGACTCGAGGAAGA

Categories of loci to which markers belong to are reported, together with their functional annotation for GAL when available. Note that GAL developed by Quéré et al. (2010; *μGH*, *mGH*, *μSL,* and *μIGF-1*) associated with growth hormone (GH), somatolactin (SL), and insulin-like growth factor 1 (IGF-1) are not reported in this table. Loci marked with an asterisk indicated loci that did not amplified in the EM samples, and were analyzed only in the AO and WM samples. Alphabetical superscripts refer to selected references in which functional role of annotated GAL markers are reported.

1 Size range established before this study.

* Locus that was not genotyped in the EM basin.

a García et al. (1998); Kultz (1998).

b O'Driscoll et al. (2008); Matchkov et al. (2008).

c Ishida et al. (1999).

d Janssens and Goris (2001).

e Wodarz (2000), Vasioukhin (2006).

Markers from SSR libraries were amplified in two multiplexes PCR (Table 2). For the first multiplex, amplification conditions have been reported in Guinand et al. (2008). The second multiplex was optimized for a final volume of 10 μL with 10× Taq buffer, 30 mmol/L MgCl_2_, 2.7 mmol/L dNTP, 10 ng of genomic DNA and 4 μmol/L of each primer. Reactions were performed on a PTC-200 (MJ Research, St Bruno, Québec, Canada) as follows: an initial denaturation at 95°C for 3 min, 35 cycles at 94°C for 45 s, annealing at the 58°C for 45 s, and 72°C for 45 s, followed by a final elongation at 72°C for 10 min. Genotyping was performed on ABI PRISM 3130xl or 3700 DNA Analyser (Life Technologies, St Aubin, France), using 5′-labeled reverse primers and the GeneScan® TM-500 LIZ Size Standard (Life Technologies) as internal size standard.

### Data analyses

**Figure 1:**
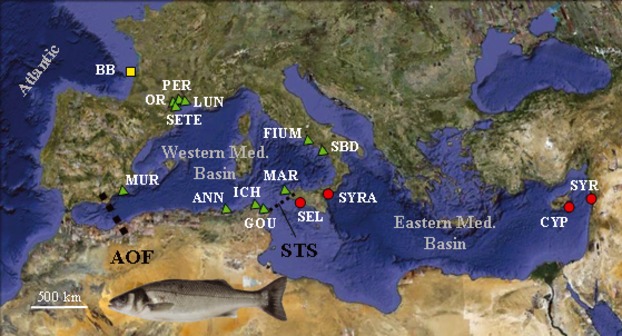
Map of sampling locations; samples are abbreviated as in Table 1. The colored symbols for samples belonging to each metapopulation will be used in other figures. Location of the Almeria-Oran Front and the Siculo-Tunisian Strait are reported.

After control for null alleles with MicroChecker (Van Oosterhout et al. 2004), all analyses – except neutrality tests – were first performed with individual samples (Fig. 1; Table 1), then with pooled samples corresponding to the three metapopulations. In order to provide descriptive summary statistics, the mean number of alleles (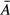), the mean unbiased expected gene diversity (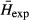; Nei 1987) were computed for each locus and each (meta)population. Significant within or among basins differences in 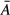 and 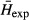 were tested using the nonparametric Mann–Whitney *U*-test implemented in the Microsoft Excel add-on, StatEL. Complementarily, genetic description of sample was assessed by computing LD. LD between pairs of loci was computed using GENETIX v4.05 (http://genetix.univ-montp2.fr/) in each sample, and tested using permutation tests (5000 permutations). Finally, deviations from Hardy–Weinberg expectations (HWE) within each original sample and within each metapopulation were investigated using 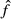 (Weir and Cockerham 1984), also using GENETIX. The null hypothesis (*f* = 0) of no significant departure from panmixia was tested by randomly permuting alleles (*n* = 1000) from the original matrix of genotypes.

Among-sample comparisons were assessed by estimating levels of population differentiation using 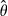, an estimator of *F*_ST_ (Weir and Cockerham 1984). Estimates of 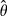 were independently computed for three data sets (i.e., the full set of 20 loci, then GAL and GIL sets). Their significance was tested according to the permutation procedure available in GENETIX and corrected for multiple tests according to Benjamini and Yukitieli (2001) false discovery rate (FDR; Narum 2006). Distance trees were inferred from Reynolds et al. (1983) coancestry genetic distance matrix [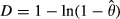] by the Neighbor procedure of the phylogenetic package Phylip 3.6 (Felsenstein 2005). Trees were visualized using Treeview (Page 1996). Following O'Reilly et al. (2004), we explored the relationship between 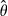 and *H*_exp_ to check for effects of size homoplasy. We specifically controlled whether GIL and GAL markers were differently affected. We further explored the possible role of selection in shaping differentiation in the full, GAL, and GIL data sets using neutrality tests developed by Beaumont and Nichols (1996; hereafter B&N and implemented in LOSITAN Antao et al. 2008, http://popgen.eu/soft/lositan/), by Vitalis et al. (2001; implemented in DETSEL Vitalis et al. 2003, http://www.genetix.univ-montp2.fr/detsel.html), and by Foll and Gaggiotti (2008; hereafter: F&G and implemented in BayeScan, http://www-leca.ujf-grenoble.fr/logiciels.htm). All tests were first performed among populations within each basin, then among the three basins except for DETSEL, specifically designed for pairwise comparisons then between each pair of basins. Outlier loci detected using several methods would be stronger candidate to the action of selection than loci detected once. The B&N and, when considered, the DETSEL tests were both performed according to *P* = 0.95 and *P* = 0.99 successively. F&G test considered a Log_10_(BF) of 1.3 and 2 (BF = Bayes Factor) corresponding to posterior probabilities of locus effects of 0.95 and 0.99, respectively.

The program MIGRATE-n v3.0 (Beerli 2008) was used to infer *M*, the migration rate, among basins only (*M* = *m*/*μ*, where *m* is the immigration rate per generation). Contrary to most *F*_st_-based statistics, MIGRATE-n provides estimation of asymmetric migrations among (meta)populations, indicating if a (meta)population is a net donor or net receiver of individuals over evolutionary times. It hence provides a picture how tension zones may function. These calculations used the Brownian mutation model and the mutation rate was considered equal for all loci (*μ* = 10^−4^) even for GAL. We used coalescent maximum likelihood (ML) based on Markov Chain Monte Carlo with Hastings Metropolis importance sampling to infer the various parameters (Beerli and Felsenstein 1999, 2001). *F*_st_ estimates among basins were used as initial parameters for the estimation of *Θ* and *M* in MIGRATE-n. For each locus, the ML was run for 10 short and five long chains with 50,000- and 10,000-recorded genealogies, respectively, after discarding the first 1000 genealogies (burn-in) for each chain. One of every 20 reconstructed genealogies was sampled for both the short and long chains. We used an adaptive heating scheme with four concurrent chains. Analyses were all performed in triplicates either on the full set of loci or on the GAL and GIL sets independently.

As a complement to estimates of population differentiation and asymmetric migration rates, we computed the probability of membership of individuals to each metapopulation (i.e., individuals should have higher probability of membership in the metapopulation they were sampled). Assignments of individuals to populations and associated probabilities were inferred with the software STRUCTURE v2.3 (Pritchard et al. 2000; http://pritch.bsd.uchicago.edu/structure.html) by setting *K* = 3 (i.e., three basins) after verification of the most likely number of independent population clusters was *K* = 3 using Evanno et al.'s (2005) Δ*K* method (details not reported). The software used a Monte Carlo Markov Chain (MCMC) Bayesian clustering method that maximizes the within-cluster Hardy–Weinberg and linkage equilibriums. The admixture model with noncorrelated allele frequencies was used for the full, GAL, and GIL data sets. A burn-in length of 50,000 iterations and subsequent 500,000 additional MCMC iterations were carried out. Individuals were assigned to clusters based on the highest probability of membership (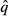-value). Five replicates were independently performed, giving reproducible results. Average 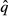-values (±1 standard deviation) of individuals to each metapopulation were hence computed from those individual assignments.

Analyses using MIGRATE-n and STRUCTURE were first performed using initial sample sizes of the three basins (Table 1), but, as sample sizes were unbalanced, we also performed analyses with 31 individuals in each basin (i.e., the number of individuals from the AO basin). Results were highly reproducible and qualitatively comparable to those obtained with the full set of individuals and will not be reported.

## Results

Numerous amplifications failures were observed for fishes from EM populations, at several loci (GAL: *DLA0081*, *mGH*; GIL: *DLA0066, DLA0073, DLA0096*). This was probably due to loss of sequence homology at PCR priming sites. Those loci were discarded from some analyses, as relevant. Therefore, genetic comparisons were performed with 20 loci (11 GAL, nine GIL), except those involving EM populations, which were only based on 15 loci (nine GAL, six GIL).

### Gene and allelic diversities

Allelic and gene diversities are reported in Table 3. There were no differences among populations within the WM or the EM basin for the whole set of loci, or for the GAL and GIL marker sets considered separately (details not reported), (20 or 15 loci, respectively). 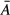 and 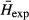 were not significantly different between the AO and WM metapopulations (*P* > 0.05 in each case; 20 loci). In contrast, 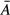 and 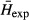 of the EM metapopulation were significantly lower than the other metapopulations for the 15 loci (*P* < 0.001). When considering the GAL and GIL marker sets independently, 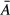 was significantly larger for GAL than for GIL in the WM metapopulation (Mann–Whitney *U*-test, *P* = 0.006). No such difference was observed between GAL and GIL in the AO and EM basins for 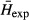 (*P* = 0.055 and *P* = 0.12, respectively).

**Table 3:** Gene and allelic diversity indices for each individual samples and metapopulations (AO, EM, WM) of sea bass considered in this study

		Multilocus			GAL			GIL		
				
Basin	Sample	*H*_*exp*_	A_r_	*f*	*H*_*exp*_	A_r_	*f*	*H*_*exp*_	A_r_	*f*
AO	GG	0.77	8.70	0.01	0.78	9.33	−0.01	0.76	7.75	0.03
WM	MUR	0.79	9.65	0.01	0.79	10.17	0.01	0.78	8.88	0.01
	OR	0.77	9.20	0.00	0.76	9.48	0.04**	0.79	8.73	0.06***
	SET	0.77	7.84	0.01	0.77	8.64	−0.01	0.78	6.75	0.03
	PER	0.77	8.61	−0.01	0.76	9.17	−0.01	0.77	7.75	0.00
	LUN	0.78	8.90	0.02	0.78	8.58	0.00	0.79	9.38	0.05
	FIUM	0.75	7.65	0.00	0.74	8.25	−0.04	0.76	6.70	−0.02
	SBD	0.77	7.36	−0.03	0.74	7.25	−0.04	0.81	7.53	−0.02
	MAR	0.76	8.13	−0.01	0.76	8.06	−0.02	0.76	8.26	0.01
	ANN	0.78	9.47	0.00	0.79	9.54	−0.02	0.77	9.38	0.03
	ICH	0.74	8.12	0.02	0.73	7.83	−0.01	0.76	8.48	−0.07***
	GOU	0.77	8.54	−0.03**	0.76	8.83	−0.03**	0.77	8.13	−0.01
	WM metapop.	0.77	8.49	0.01	0.76	8.71	−0.01	0.78	8.18	0.01
EM	SEL	0.68	5.24	−0.07	0.69	6.10	−0.08*	0.66	4.19	−0.05
	SYRA	0.60	3.93	−0.22***	0.61	4.20	−0.24***	0.56	3.25	−0.18
	CYP	0.61	4.13	−0.06	0.67	4.48	−0.06	0.57	3.62	−0.07
	SYR	0.66	5.21	−0.08	0.67	5.82	−0.06	0.62	4.03	−0.06
	EM metapop.	0.64	4.62	−0.07***	0.67	5.15	−0.07***	0.61	3.76	−0.06

AO, Atlantic Ocean; EM, Eastern Mediterranean; WM, Western Mediterranean; GAL, gene-associated loci; GIL, gene-independent loci.

Multilocus estimates of *f* are also provided. *P < 0.05; **P < 0.01; ***P < 0.001.

### Hardy–Weinberg equilibrium and linkage disequilibrium

After corrections for multiple tests, no significant departure from HWE was detected for any individual samples belonging to the AO and WM basins, whatever data were considered (not reported). HWE was also respected after pooling the WM samples into a single metapopulation (all markers: 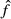 = 0.01, NS; GAL: 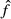 = −0.01, NS; GIL: 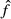 = 0.01, NS; Table 3). For the EM populations, there was a significant heterozygote excess over the 15 loci in the SYRA (Syracuse) population (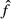 = −0.22; *P* < 0.001), while HWE was respected in other individual samples. This induced a significant heterozygote excess in the EM metapopulation (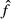 = −0.07; *P* < 0.001). It was mostly due to GAL markers in the SYRA and SEL (Selinunte) populations (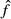 = −0.24, *P* < 0.001; 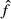 = −0.08, *P* < 0.05, respectively). No significant departure from HWE was detected in the EM metapopulation for the multilocus GIL marker set (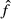 = −0.06; NS; Table 3).

Because larger sample sizes prevent detection of spurious genotypic associations among loci, analysis of LD was primarily performed at the metapopulation level for Mediterranean samples (i.e., AO consists of a single sample). After correction for multiple tests, there were only three significant cases of LD in the WM metapopulation. In the EM metapopulation, 12 significant cases of LD were found, that involved only four loci: two GIL and two GAL markers (*DLA0068*, *DLA0086*, *DLA0070* and *DLA0089*).

### Relationships between 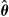 and gene diversity

Relationship between 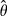 and *H*_exp_ within WM and EM revealed no significant negative correlations (WM_[20 loci]_: *R*^2^ = 0.062, NS; EM_[15 loci]_: *R*^2^ = 0.129, NS; Fig. S1). There were no significant correlations by pairwise metapopulation comparison, AO–WM, AO–EM and WM–EM, nor comparison among the three metapopulations (AO–WM_[20 loci]_: *R*^*2*^ = 0.158, NS; AO–EM_[15 loci]_: *R*^*2*^ = 0.052, NS; WM–EM_[15 loci]_: *R*^*2*^ = 0.043, NS; three metapopulations_[15 loci]_: *R*^*2*^ = 0.028, NS). When present, homoplasy did not significantly affect data and these results legitimize further interpretation in terms of differential gene flow and/or selection.

### Genetic differentiation within the WM and EM metapopulations

Within the WM basin, no significant differentiation was detected (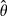 = 0.004, NS). There was no differentiation for the GIL or the GAL data separately (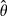 = 0.001, NS; 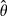 = 0.004, NS, respectively). Within the EM basin, there was significant differentiation only for the GAL data (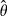 = 0.049, *P* < 0.01), but not for the full or GIL data (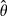 = 0.021, NS; 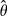 = −0.006, NS, respectively).

### Genetic differentiation among basins

**Figure 2:**
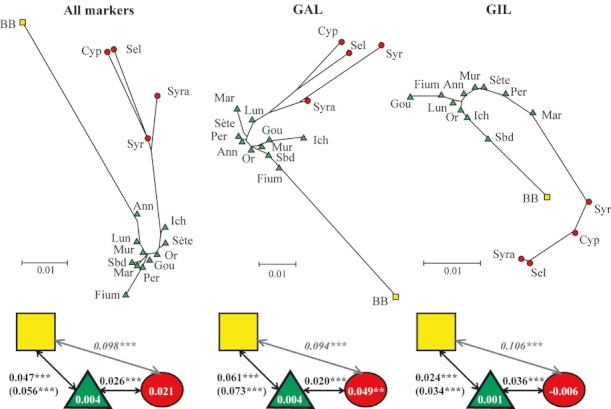
Reynold's distance trees for each marker set used in this study (all markers [15 loci]: GAL [9 loci] and GIL [6 loci]). Figures located at the bottom of each tree summarized patterns of genetic differentiation among samples within metapopulation (no estimate for Atlantic Ocean [AO] as one unique sample was considered in this metapopulation; Table 1), and among metapopulations as estimated by 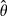 for each marker set. In the case of the AO–Western Mediterranean, 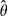 values are also reported in brackets for the 20 loci marker set, then for the set of 11 and 9 GAL and GIL marker sets, respectively. Samples are abbreviated as in Table 1 and colored symbols as in Fig. 1. **P < 0.01, ***P < 0.001.

Reynolds' distance trees based on the 15 locus data clearly partitioned individual samples into three basins (Fig. 2). Levels of differentiation were generally significant and dependent on the pairwise basin comparison and markers used (GAL or GIL; Fig. 2). The differentiation between AO and WM was greater than between WM and EM, whatever data considered (Fig. 2).

**Figure 3:**
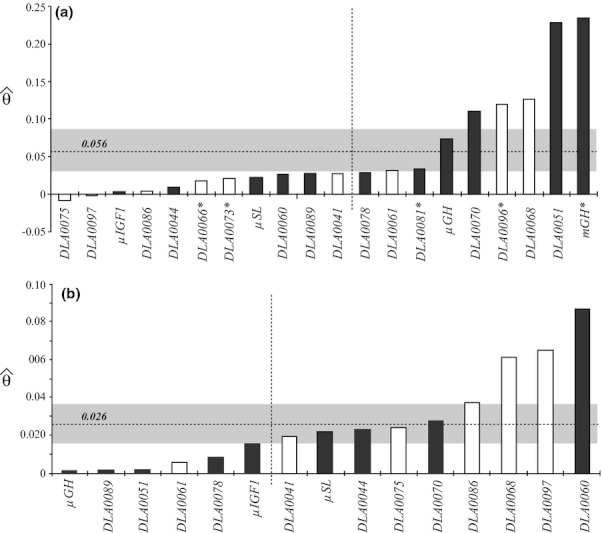
Estimates of 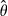 observed at each individual locus for (a) the Atlantic Ocean–Western Mediterranean (WM) comparison (20 loci), and (b) the WM–Eastern Mediterranean (EM) comparison (15 loci). Loci are arranged according to increased values Of 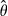 in each comparison. Horizontal lines indicate mean values of 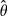 observed in each comparison (shaded area = 95% CI). Vertical lines indicate which individual loci were found significant (on the right) compared to those that were not (on the left) for each comparison. Open and black boxes indicate GIL and GAL, respectively. Asterisks indicate loci absent in the WM–EM comparison.

The individual values of 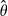 for each locus in the AO–WM and the WM–EM comparisons are reported in Figure 3A,B, respectively. Between AO and WM (20 loci used), five loci had 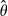 > 10%, including locus *mGH* where 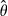 ≍ 25%. The multilocus differentiation estimator 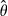 was 0.056 (*P* < 0.001) with significant differentiation at nine markers (GAL: six markers; GIL: three markers, Fig. 3A). The GAL data had higher values of 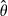 than the GIL data (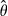 = 0.073 vs. 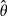 = 0.034; both *P* < 0.001; Fig. 2). Using 15 loci, levels of genetic differentiation were significant between WM and EM (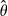 = 0.026; *P* < 0.001), with significant individual estimates of 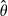 at nine of the 15 loci (GAL: four markers; GIL: five markers; Fig. 3B). Contrary to the AO–WM comparison, differentiation at the GIL markers was greater than at the GAL markers in the WM–EM comparison (respectively, 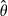 = 0.036 and 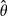 = 0.020, both *P* < 0.001; Fig. 2).

### Patterns of genetic differentiation

**Figure 4:**
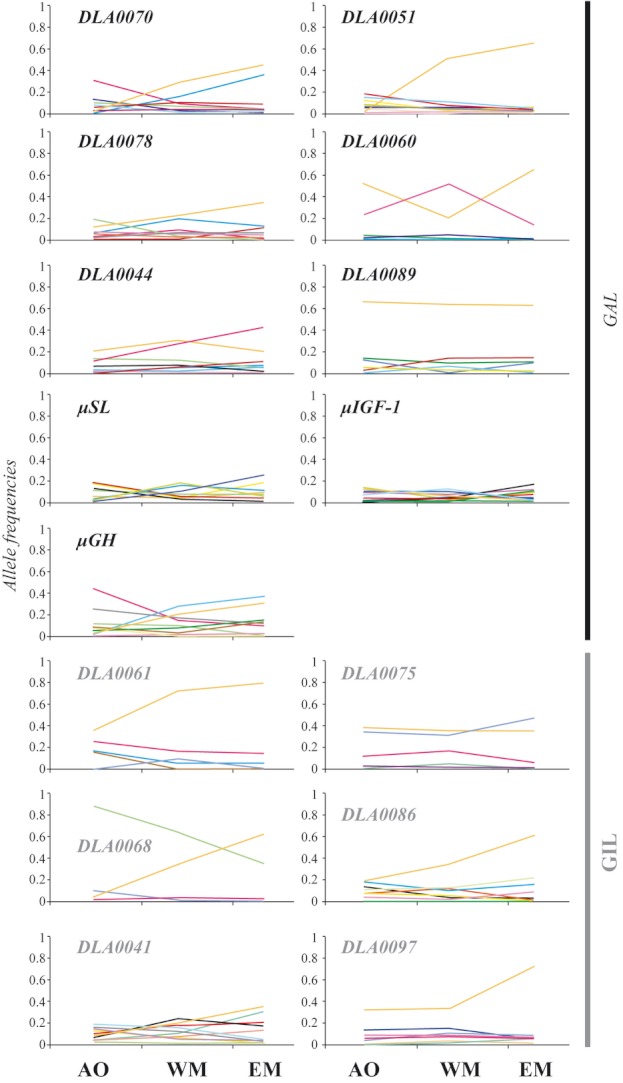
Variation of allele frequencies observed at each individual locus among sea bass metapopulations. For each marker, colored lines represent variation for a single allele. Because of size of the panels associated to loci, all alleles might not be visible at each locus. Metapopulations are abbreviated as in Table 1.

There was wide variability in spatial patterns of allelic frequency changes (Fig. 4), including cases where allele frequencies were (1) almost identical in all metapopulations, reflecting either high gene flow, shared ancestral polymorphisms, or general selective sweeps at these loci (e.g.*, DLA0089*, *μGH*, resulting in nonsignificant 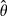 values in Fig. 3B), (2) identical in two adjacent metapopulations (AO–WM or WM–EM) then marking a sharp break at one of the transition zones (e.g.*, DLA0051, DLA0097*), or (3) in an apparent east–west gradient of allele frequencies (e.g.*, DLA0068*). Those latter two classes of loci naturally coincided with loci achieving higher levels of differentiation (Fig. 3). Locus *DLA0060* appeared as a particular case with almost identical allele frequencies for the metapopulations located at each side of the distribution but drastic change in frequency for the most frequent alleles in the central metapopulation.

### Population assignment and migration

**Figure 5:**
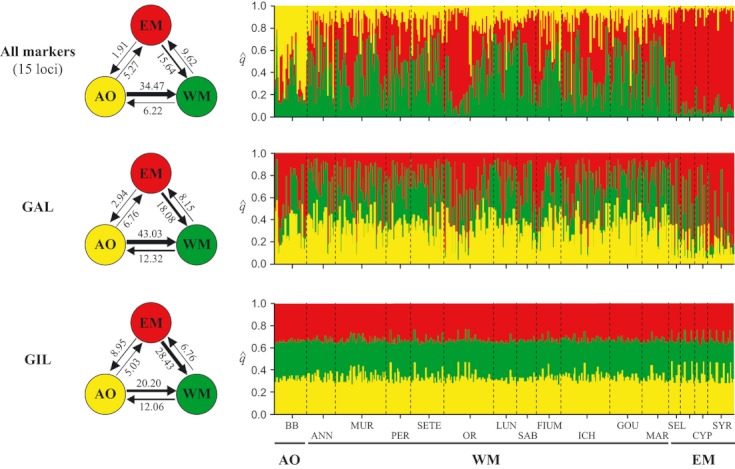
Summary of results obtained by MIGRATE-n regarding the estimation of number of migrants among sea bass metapopulations for each set of markers considered in this study (15 loci data set; left panel), and STRUCTURE regarding probability of membership of each individual to one of the *K* = 3 sea bass metapopulations considered in this study. Thickness of arrows is broadly proportional to estimated number of migrants. Samples and metapopulations are abbreviated as in Table 1.

Analyses regarding assignment were initially performed for the 15 markers in all metapopulations, then only using GAL (*n* = 9; *DLA0044, DLA0051, DLA0060, DLA0070, DLA0078, DLA0089, μGH, μIGF1, μSL*) or GIL (*n* = 6; *DLA0041, DLA0061, DLA0068, DLA0075, DLA0086, DLA0097*; Fig. 5). Unfortunately, some loci with a dramatically high 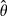 between AO and WM could not be considered because they failed to amplify in EM (e.g.*, mGH*; Fig. 3A). This lowered the probability of assignment for AO and WM individuals. Nevertheless, better mean average assignment to their population of origin was achieved for AO and EM individuals (mean 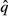-value = 0.548 ± 0.156 and 0.868 ± 0.151, respectively) than for the WM individuals (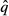 = 0.326 ± 0.206; WM individuals were in average better assigned to the EM cluster: 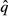 = 0.487 ± 0.291 when using all loci). This suggested higher introgression in the WM compared with the other basins (Fig. 5), and correlated well with lower all-marker genetic differentiation estimated at the STS compared to the AOF (Fig. 2). When analyzing the GAL, only EM individuals were better assigned on average to their actual population of origin (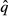 = 0.596 ± 0.223), while correct assignment was far lower for other metapopulations (AO: 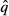 = 0.417 ± 0.117; WM: 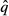 = 0.370 ± 0.134). When the GIL was used and whatever the metapopulation considered, probabilities of correct assignment did not differ significantly from 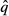 = 0.33 (details not reported). It has to be emphasized that results for GIL in this analysis did not indicate lower intrinsic relevance of GIL to assign individual, nor increased gene flow for this class of loci, but were primarily due to the low number of GIL in this particular analysis (*n* = 6) with only one locus demonstrating genetic differentiation among each pair of basin (*DLA0068*; Fig. 3). At the scale of the three metapopulations, it resulted that those six loci collectively had significant but low average 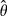 = 0.031 (*P* < 0.001), resulting in poor ability to successful individual assignment. Using simulated data sets in a five deme system, Latch et al. (2006) reported an average value of *θ* = 0.030 as a lower bound to consistently assign individuals to a subpopulation (i.e., with a probability distinct from equiprobability). Empirical 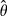 observed in sea bass is very close from this lower bound, easily explaining poor assignment capacities with this data set.

Individual WM samples located close to the STS (e.g.*,* GOU, MAR; Fig. 1) present a compound allele pattern slightly closer to that of individuals from the EM samples than to WM samples located further west (e.g., MUR) for all assignments, either performed over the full data set or with the random subsets of 31 individuals. Despite this, WM individuals from western and eastern WM samples were not assigned significantly differently from EM samples over five replicates, whatever the number and class of markers considered (details not reported).

Maximum-likelihood estimates of migrations using MIGRATE-n revealed asymmetric exchange from AO to WM with a approximately threefold to fivefold difference in number of successful migrants when using GAL and all markers (GAL: *M*_AO→WM_ = 43.03 vs. *M*_WM→AO_ = 12.32; all markers: *M*_AO→WM_ = 34.47 vs. *M*_WM→AO_ = 6.22; Fig. 5). This asymmetry of migration between AO and WM was inferior to two for the GIL data set (Fig. 5). Between WM and EM, the asymmetry was also estimated with a higher number of alleles from EM migrating toward WM. The extent of the asymmetrical exchange was however lower when considering all markers (*M*_EM→WM_ = 15.64 vs. *M*_WM→EM_ = 9.62), or the GIL and GAL data sets separately (Fig. 5). Results of MIGRATE-n therefore indicate greater introgression in WM than in other metapopulations. That is, WM received more alleles by migration than they provided to the other two metapopulations, suggesting it may represent a hybrid swarm separated by two tension zones at the AOF and STS where migration of genes are asymmetrically filtered. It was however expected that lower estimates of genetic differentiation should translate in higher estimated number of migrants at the STS. This point has to be investigated further.

### Neutrality tests

Neutrality tests were initially performed on individual samples within the WM and EM metapopulations, respectively. DETSEL was not used at this scale because of too many, not fully independent pairwise comparisons. In WM, no congruence between the B&N or the F&G tests was detected among potentially selected loci when using *P* = 0.95 (or Log_10_(BF) = 1.3). No locus was significant for a more stringent probability or BF (not reported). Indeed, B&N indicated that three loci were outliers and hence could potentially be under selection (*μSL*, *DLA0066* and *DLA0070*) in WM, while F&G detected eight different loci (*DLA0044*, *DLA0068*, *DLA0081*, *DLA0089*, *DLA0096*, *μIGF-1*, *μGH,* and *mGH*). In EM, F&G did not detected potentially selected loci whatever the Log_10_(BF) considered, while B&N detected three loci at *P* = 0.95 (*μSL*, *μGH,* and *μIGF-1*; none at *P* = 0.99). It must be noted that those loci demonstrated the highest levels of differentiation among EM samples (details not shown). As results of neutrality tests failed to converge, the reality of nonneutrality within metapopulations is open to question.

Results were slightly more conclusive when performing neutrality tests among metapopulations. In the AO–WM comparison, the three tests did not provide similar results, but all indicated locus *DLA0068* (GIL) as a possible outlier (Table 4). For the WM–EM comparison, B&N and DetSel detected the *DLA0060* (GAL), *DLA0068,* and *DLA0097* (GIL) loci as outliers for a minimal probability level of *P* = 0.95. F&G also detected three outlier loci for Log_10_ (BF) > 1.3: *DLA0068*, *μIGF-1* and *μSL* (Table 4). Locus *DLA0068* was detected by all three tests and was the locus with the sharpest pattern of graded variation across metapopulations (Fig. 4).

**Table 4:** Summary of loci found as being potentially selected at the metapopulation level in each outlier detection selective test used in this study (B&N: Beaumont and Nichols 1996; DetSel: Vitalis et al. 2001; F&G: Foll and Gaggiotti 2008)

		Neutrality Test		
		
	Locus	B&N	DetSel	F&G
**A:** AO vs. WM	*DLA0051*	0.993	>0.99	–
	*DLA0060*	–	>0.95	–
	*DLA0061*	–	>0.95	0.992
	***DLA0068***	**0.973**	**>0.99**	**1.0**
	*DLA0070*	0.995	>0.95	–
	*DLA0075*	0.999	–	0.976
	*DLA0096*	0.958	>0.99	–
	*DLA0097*	0.999	–	0.952
	*mGH*	0.978	>0.99	–
	*μIGF1*	0.997	–	1.0
	*μSL*	0.967	–	0.983
**B:** WM vs. EM	*DLA0060*	0.993	>0.95	–
	***DLA0068***	**0.964**	**>0.99**	**0.982**
	*DLA0097*	0.996	>0.99	–
	*μIGF1*	–	–	1.0
	*μSL*	–	–	0.997

AO, Atlantic Ocean; EM, Eastern Mediterranean; WM, Western Mediterranean.

Locus *DLA0068* found as an outlier in each test is indicated in bold.

## Discussion

Genetic differentiation between Atlantic and Mediterranean populations of marine organisms is usually interpreted as the signature of secondary contact between two formerly allopatric lineages, occurring since the last glacial maximum (Borsa et al. 1997; Patarnello et al. 2007; but see, e.g., Grant 2005; Limborg et al. 2012). Accordingly, support for two distinct mtDNA clades, or nuclear differentiation, between Atlantic and Mediterranean populations at the AOF is numerous in marine organisms (Patarnello et al. 2007). When differentiation at the AOF is not demonstrated (e.g., Bargelloni et al. 2003), differentiation is generally detected in another area of the Mediterranean, including the STS and some other locations (e.g., south of Peloponnesus; Patarnello et al. 2007). The originality of sea bass resides in their partition into three metapopulations, separated by two areas of reduced genetic connectivity: the AOF and the STS (Naciri et al. 1999; Bahri-Sfar et al. 2000; Lemaire et al. 2005). To our knowledge, reports of two locations of reduced connectivity within the Mediterranean are pretty scarce, apart from the Black Sea (but see Rolland et al. 2007; Zulliger et al. 2009; Limborg et al. 2012). In sea bass, the hypothesis of a purely physical barrier shaping variation at the AO–WM transition has been seriously reassessed by Lemaire et al. (2005), who demonstrated a tension zone at this location. By using nuclear markers (GAL and GIL), we expanded on the study by Lemaire et al. (2005) to dissect the sea bass' patterns of genetic variation further.

### Patterns of genetic differentiation and support for a tension zone at the Siculo-Tunisian strait

This study did not change the perception of sea bass genetic structure within each basin at nuclear loci (i.e., no differentiation inside the WM basin but some differentiation within the EM basin; Garcia de León et al. 1997; Naciri et al. 1999; Bahri-Sfar et al. 2000; Castilho and Ciftci 2005), nor the existence of three metapopulations. Nevertheless, estimates of differentiation among successive pairs of basins were larger than previously estimated with fewer microsatellite loci. The differentiation between the AO and WM basin reported in this study (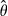
_[20 loci]_ = 5.6%) was more than twice that provided by Naciri et al. (1999) over six loci (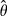 ≍ 2.1%). Estimates of differentiation at the WM–EM transition zone were also higher (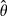
_[15 loci]_ ≍ 2.6%) than those reported by Bahri-Sfar et al. (2000) (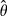 ≍ 1.4%; six loci). Results hence confirmed a stronger nuclear genetic barrier at the AOF than at the STS. This pattern was also demonstrated for mtDNA in sea bass (Lemaire et al. 2005; R. Rondon, E. Desmarais, F. Bonhomme and B. Guinand, unpubl. results), but also in sprat (Limborg et al. 2012). Using far less nuclear markers, Rolland et al. (2007) suggested on the contrary that differentiation should be stronger at the STS compared with the AOF for the common sole (*Solea solea*).

GAL and GIL markers may both be involved in overall differentiation and/or differentiation among adjacent metapopulations, but the extent of differentiation depended on the particular comparison. Indeed, GAL that are located closer from or even within genes consistently demonstrated in average reduced genetic connectivity compared GIL at the AO–WM transition (20 loci; 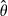
_GAL_ = 7.3%; 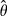
_GIL_ = 3.4%). However, a reverse relationship was found at the WM–EM transition (15 loci; 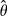
_GAL_ = 2.0%; 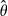
_GIL_ = 3.6%). This second observation hence partly conflicts with those reported in other studies in which gene-associated markers had, on average, better discriminatory power than independent markers (e.g., Vasemägi et al. 2005; Yatabe et al. 2007; Shikano et al. 2010; Vilas et al. 2010).

Besides differentiation, it is also interesting to note that significant LD and heterozygote excess was concentrated in a few samples. Heterozygote excess was especially marked for GAL at the WM–EM transition on both sides of Sicily: for the SEL and GOU samples, and at the Messina canal for the SYRA sample. LD only occurred in the SEL and SYRA samples from the EM basin. This observation is unlikely to be due to low sizes of SYRA and SEL samples, as others with similar sizes did not demonstrate any heterozygote excess and very few cases of LD. The GOU, SEL, and SYRA samples are all located at the WM–EM transition, suggesting that heterozygote excess and/or LD may be the signature of hybridization among parental pools at the STS, as already demonstrated by Lemaire et al. (2005) at the AOF. There, significant LD was only found in the sample closest to the AOF. In this study, we report a nearly identical situation at the STS (but note that the MAR sample also located close to the front did not show significant LD and/or heterozygote excess, in line with previous results by Naciri et al. (1999) and Bahri-Sfar et al. (2000) who also used this sample). Results are in agreement with the definition of a tension zone rather than with strict isolation by distance or parapatric differentiation. By theoretical modeling, Irwin (2012) has shown that genetically differentiated haploid clades arising from local adaptation along an environmental gradient can arise in a parapatric fashion under certain conditions and persist in a metapopulation even in the presence of moderate gene flow. Nevertheless, extension to multilocus diploid cases in high gene flow species has not been done, so it seems reasonable to stick to the less costly hypothesis of historical differentiation followed by secondary contact. If there was a single reason for this more parsimonious explanation, this would be at least because paleobiogeography indicates that favorable ecological zones have indeed been fragmented several time during the glacial maximums, notwithstanding other theoretical considerations about all the conditions required for the progressive building up of multilocus differentiation in presence of gene flow in highly dispersing species. We hence can reasonably be confident that the encounter of differentiated genetic backgrounds in the sea bass is a likely possibility as demonstrated for other marine organisms with large dispersal capabilities (e.g., bivalve mollusks, Nikula et al. 2008).

### Spatial patterns support tension zones, but rarely selection at target loci

In addition to LD and/or heterozygote excess, spatial patterns of allele frequency changes are in accordance with the prediction of a tension zone at both the AO–WM and WM–EM transitions. The theory predicts that allele frequency changes are more or less pronounced depending on the marker investigated, with steeper transitions for loci closer to “isolation genes” (Barton and Hewitt 1985). In this study, some markers present no change in allele frequency, reflecting either one unconstrained high neutral gene flow, a selective sweep of one uniformly advantageous allele or the inheritance of an ancestral state where allele frequencies were already homogeneous. On the other hand, some loci show relatively sharp changes at AO–WM transition as already shown for mtDNA (Lemaire et al. 2005), or at the WM–EM transition, which has been less extensively investigated (Bahri-Sfar et al. 2000). A vicariant event that produced at least two separate Atlantic and Mediterranean metapopulations of sea bass during one of the quaternary climatic oscillations will have led to the emergence of isolation genes. These endogenous barriers influenced many other loci by indirect (hitchhiking) selection at genomic scales, depending on their strength and the amount of recombination (Barton and Hewitt 1985; Piálek and Barton 1997; Slatkin and Wiehe 1998). According to general hybrid zone models, we postulate that barriers have been trapped at various locations which correspond to areas of reduced density and/or environmental change, such as the AOF and STS. A rationale of this phenomenon is provided in Bierne et al. (2011), including a review of marine examples (see also Luttikhuizen et al. 2012).

Locus *DLA0060* (GAL; located in intron one of the bestrophin three gene) presents a distinct pattern of spatial allele frequency compared to other loci, with the two most frequent alleles at higher frequencies in WM than in the marginal AO and EM metapopulations In the present case, we cannot distinguish whether locus *DLA0060* was linked to an “endogenous” locus whose variation in allele frequency reflects genomic incompatibilities, or to an “exogenous” locus whose variation reflects local adaptation, both fitting with the coupling hypothesis of Bierne et al. (2011).

In hitchhiking models of genetic variation, direct selection on markers is not needed (Piálek and Barton 1997; Bierne et al. 2003). In sea bass, neutrality tests were unable to identify loci with clear footprint of selection, except *DLA0068* (GIL). This locus presents a steeper gradient of allele frequencies than other loci, with a nearly fixed allele in the AO sample and an alternative one that predominates in the EM sample. This marker was not clearly associated with a gene. It is situated at approximately 4 kb from the 3′ end of a Retinal G Protein Coupled Receptor gene (*RGR*) on one side and approximately 6 kb from the 5′ end of the CDS of a solute carrier gene (*SLC18A3* also known as *VAChT*, e.g., Weihe et al. 1996; Karila et al. 1998) on the other. We cannot therefore reach firm conclusions about its functional importance.

### A hybrid swarm in the Western Mediterranean?

The WM contains strongly introgressed individuals, but also receives migrants from other basins. Taken together, this indicates that the WM metapopulation may have a dual origin from both AO and EM nuclear genomes, which have introgressed into each other and led to the emergence of a new reproductive unit, known as a hybrid swarm (Arnold 1993; Jiggins and Mallet 2000). The absence of LD among loci in each WM sample, except the GOU sample located close to the STS, also supports this hypothesis of a sea bass hybrid swarm. Interestingly, Schunter et al. (2011) recently reported a similar result in the comber *Serranus cabrilla* within the WM basin. In their case, combers from locations located between the two recognized fronts for this species (AOF and the Ibiza Channel) were significantly more introgressed than individuals from populations on the external sides of each front, although the presence of tension zones was not investigated. Our results on sea bass are also comparable to experimental studies by Edmands et al. (2005) on the copepod *Tigriopus californicus*. After approximately 15 generations of mixing, Edmands et al. (2005) found more hybrids than expected, together with heterozygote excess. These experimental results conform to the situation observed in wild sea bass near tension zones, as heterozygote excess and LD were recorded at the STS and AOF (this study and Lemaire et al. 2005, respectively). Overall, the results suggest that the WM metapopulation of sea bass could result from hybrid swarming, that is separated from parental forms by two distinct tension zones.

Hybrid swarms are poorly documented in marine organisms (McMillan et al. 1999; Roques et al. 2001; Roberts et al. 2010) by comparison with terrestrial or freshwater taxa (e.g., Arnold 1997; Nolte et al. 2009; Stemshorn et al. 2011). They may be restricted to narrow marine zones (estuaries; see Roberts et al. 2010) or extend over larger areas (Ayres et al. 2004; Strelkov et al. 2007; Nikula et al. 2008). Strelkov et al. (2007) analyzed patterns of genetic variation in a hybrid swarm of *M. balthica* that extends over hundreds of kilometers in the Norwegian, Barents, and Pechora seas. The samples within this swarm also showed a component of regional differentiation that was not observed in sea bass: within WM, no differentiation was detected and there was no significant indication of differential introgression with AO and EM individuals for samples located to the west or the east.

The random coupling of exo- and endogenous incompatibilities hypothesized by Bierne et al. (2011) can elicit emergence of distinct tension zones between successive fragmented patches of habitat. Using a two-locus stepping-stone model of gene flow with overdominance, Goodisman and Crozier (2001) also demonstrated that two spatially distinct tension zones can emerge between formerly isolated populations, giving rise to an additional central population. Goodisman and Crozier (2001) also demonstrated that LD accumulated at the two tension zones separating the three successive populations, while it disappeared in the central population, as observed in sea bass in the WM basin.

Although sea bass population structure and pattern of population differentiation conform to the expectations of some models, it is essential to acquire further data to investigate the hypothesis of a hybrid swarm in the WM. Such caution particularly derives from statistical considerations. This includes the discrepancy between estimated levels of differentiation and estimated number of migrants at the STS. It was expected that lower genetic differentiation should translate in higher number of estimated migrants, but this trend was not observed possibly casting doubt on this particular result. Furthermore, numerous methods for analysis of hybridization processes have emerged over recent years. They range from identification of F_1_ to F_X_ hybrids and backcrosses (e.g.*,* Anderson and Thompson 2002), to evaluating the fitness effects of alleles transferred across different genetic backgrounds (i.e., introgression; Gompert and Buerkle 2009), to genetic diffusion models (Stemshorn et al. 2011). Methods aim to analyze introgression (admixture) patterns together with the dynamics and history of introgression (Nolte and Tautz 2010; Lamaze et al. 2012). To derive consistent outputs, however, they require clear recognition of parental genotypes (Nolte et al. 2006, 2009; Gagnaire et al. 2011; Luttikhuizen et al. 2012). This is typically not observed in sea bass, where parental genotypes (AO, but also EM metapopulations) are both significantly introgressed, hence limiting the capacity for reliable inference. High throughput genome-wide genotyping will probably provide the statistical power to disentangle recent from old and shared from unshared polymorphism across transition zones in sea bass.

## Supporting Information

**Figure S1.** Single-locus estimates of *F*_st_ as estimated by  (mean ± 95% CI) vs. expected heterozygosity for (a) the 20 loci used for the Western Mediterranean samples, and (b) the 15 loci used for the Eastern Mediterranean samples. Relationship between  and *H*_exp_ revealed no significant negative correlations (Western Mediterranean: *R*^2^ = 0.062, NS; Eastern Mediterranean: *R*^2^ = 0.129, NS). Open and filled diamonds indicate GIL and GAL, respectively. Labels of individual loci are not reported.
