# Supplementary figures and images for: Gene flow at major transitional areas in sea bass (Dicentrarchus labrax) and the possible emergence of a hybrid swarm
Source: Ecol Evol. 2012 Nov 8;2(12):3061–78. doi: 10.1002/ece3.406 (PMC3539001; doi:10.1002/ece3.406)

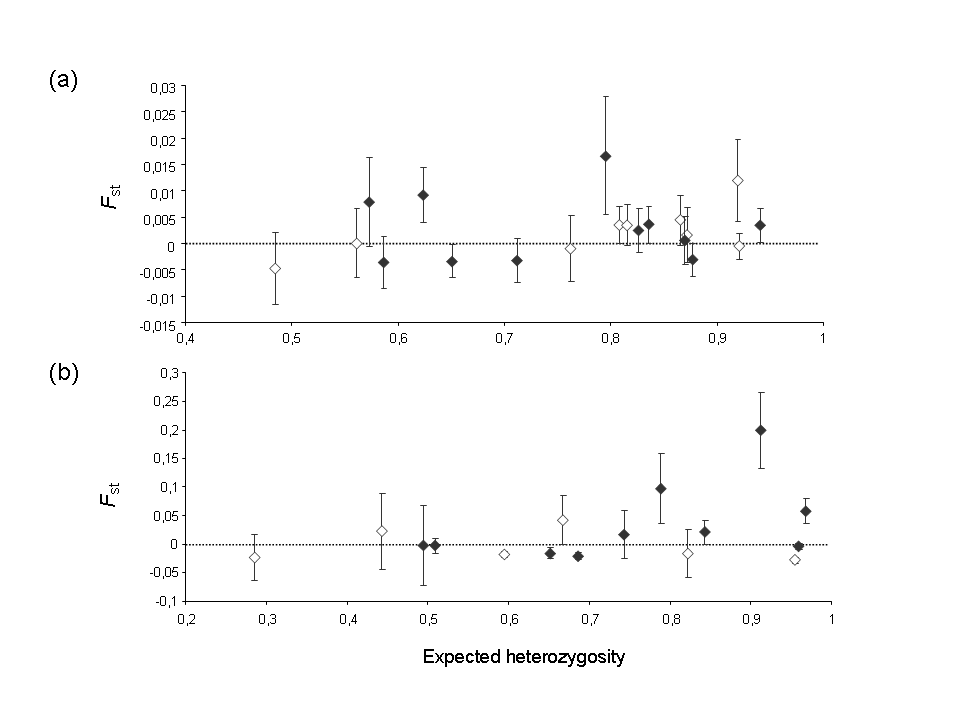

Supplement: Supplementary file 1 [file ece30002-3061-SD1.png]
